# Iron deficiency among low income Canadian toddlers: a cross-sectional feasibility study in a Community Health Centre and non-Community Health Centre sites

**DOI:** 10.1186/s12875-018-0848-9

**Published:** 2018-09-24

**Authors:** Imaan Bayoumi, Patricia C. Parkin, Gerald Lebovic, Rupa Patel, Kendra Link, Catherine S. Birken, Jonathon L. Maguire, Cornelia M. Borkhoff

**Affiliations:** 1 Department of Family Medicine, Queen’s University, 220 Bagot St., P.O. Bag 8888, Kingston, ON K7L5E9 Canada; 2 Division of Paediatric Medicine and the Paediatric Outcomes Research Team (PORT), Hospital for Sick Children, Toronto, ON Canada; 3 Sick Kids Research Institute, Toronto, ON Canada; 4 Institute of Health Policy, Management and Evaluation, University of Toronto, Toronto, ON Canada; 5 Division of Paediatric Medicine, Department of Paediatrics, Faculty of Medicine, University of Toronto, Toronto, ON Canada; 6 Applied Health Research Centre (AHRC), Toronto, Canada; 7 Li Ka Shing Knowledge Institute, St. Michael’s Hospital, Toronto, ON Canada; 8 Kingston Community Health Centres, Kingston, Canada; 9 Department of Paediatrics, St. Michael’s Hospital, Toronto, ON Canada; 10 Department of Nutritional Sciences, Faculty of Medicine, University of Toronto, Toronto, ON Canada

**Keywords:** Early childhood, Iron deficiency, Social determinants of health

## Abstract

**Background:**

Iron deficiency in early childhood has been associated with poor developmental outcomes. Little is known about the nutritional health of young children receiving care at Canadian Community Health Centres (CHCs). Our objectives were to describe iron deficiency among toddlers at an Ontario CHC, to compare young children attending CHCs and non-CHCs, and assess the feasibility of conducting research on children in CHC settings.

**Methods:**

One CHC, Kingston Community Health Centres (CHC) with two clinical sites and one community programming site was added to the nine non-CHC pediatric and primary care clinics in the existing TARGet Kids! research network. A cross-sectional feasibilitystudy was conducted.and. Healthy children, ages 12–36 months were Enrolle*d. iron* deficiency without inflammation (ferritin< 14 μg/L and CRP < 10 mg/L) and serum ferritin were assessed. Adjusted multivariable regression analyses were used to evaluate an association between CHC enrolment and iron status.

**Results:**

The CHC cohort (*n* = 31) was older, had lower household income, lower maternal education, higher nutrition risk scores, higher cow’s milk intake, shorter breastfeeding duration and higher prevalence of unhealthy weights compared with the non-CHC cohort (*n* = 875). There was no association between CHC status and serum ferritin (difference in median serum ferritin 4.78 μg/L, 95% confidence interval [CI] -2.5, 14.3, *p* = 0.22) or iron deficiency (OR 0.55, 95% CI 0.11, − 2.73, *p* = 0.46) using multivariable linear and logistic regression, respectively.

**Conclusion:**

Despite differences in sociodemographic variables, we did not detect a difference in iron status between toddlers enrolled at CHCs compared to non-CHC settings. Further research is needed to understand the health effects of poverty generally, and iron deficiency specifically among children receiving care at CHCs.

## Background

Healthy term infants are born with adequate stored iron. However, without ongoing adequate intake, non-anemic iron deficiency (NAID) can develop and progress to iron deficiency anemia (IDA), which has been associated with long lasting and potentially irreversible impaired mental and psycho-motor development [1–4].

Infants and toddlers are vulnerable to iron deficiency because their natural diet tends to be low in iron and their needs are high due to rapid growth. There are no nationally representative data but regional studies suggest a prevalence among young Canadian children of 12% or higher for NAID and 1.5% or higher for IDA, [5] which is comparable to U.S. findings [6, 7].

*TARGet Kids!* (www.targetkids.ca), an innovative practice-based research network has recruited more than 10,000 young children, mostly located in Greater Toronto area, Canada during their regularly scheduled health maintenance visits with their primary care providers since 2008. Iron deficiency prevalence and risk factors have been studied in this cohort [8–15].

In the province of Ontario, Canada, 74 publically funded Community Health Centres (CHCs) provide primary health care to over 30,000 young children under age 6 years. Children accessing health care from CHCs may be at increased risk for poor health outcomes due to a high prevalence of poverty and low parental educational attainment [16–18]. CHCs have been identified as an ideal setting for studying child health, however to date there has been no previous research conducted on young children attending CHCs [19].

The Rourke Baby Record, a Canadian evidence-based guide endorsed by the Canadian Paediatric Society and the College of Family Physicians of Canada for health supervision for well child care recommends screening high risk infants for anemia, but makes no recommendation on screening for iron deficiency in young children [20].

If children attending CHCs are at increased risk for iron deficiency, they could be targeted for screening. Therefore, our primary objective was to describe iron deficiency among toddlers attending CHCs through the addition of a CHC site to the TARGet Kids! network; and our secondary objective was to determine whether CHC attendance, was associated with iron deficiency in early childhood.

## Methods

We performed a multi-centre cross-sectional feasibility study. We recruited the CHC sample from December 2013 to August 2014 from Kingston Community Health Centres (KCHC) including two clinical sites (North Kingston Community Health Centre and Napanee Area Community Health Centre) and a community site (Better Beginnings for Kingston Children) which offers programs for families with children age 0–5 years living in north Kingston, including prenatal education, home visiting and parenting programs. Parents of potentially eligible children were invited to participate by letter, with a follow up phone or text message. We attempted to remove barriers to participation by providing transportation, childcare, snacks and a $10 grocery card.

The non-CHC sample comprised children participating in TARGet Kids! recruited by study personnel embedded at 9 participating pediatric and primary care practices in Toronto, Canada between June 2008 and March 2015. The TARGet Kids! cohort profile has been previously described [8]. Data for the non-CHC sample were abstracted from the TARGet Kids! database.

We enrolled healthy children ages 12 to 36 months, with their guardian’s written consent. Children were ineligible to participate if they had a previously diagnosed developmental disorder, genetic, chromosomal or syndromic condition, chronic medical condition (other than asthma and allergies), were born prematurely (gestational age less than 35 weeks), birth weight less than 2500 g, had an acute illness such as a viral illness or whose parent was unable to communicate in English. For the purpose of this study, children were included if they had complete data on outcome variables including serum ferritin, and C-reactive protein (CRP). Children with CRP ≥10 mg/L were excluded since this elevation may indicate acute systemic inflammation [21, 22]. If a child’s record contained data from multiple visits, the first visit with complete data was used for analysis.

Research ethics approval was granted by Queen’s University Health Sciences Research Ethics Board (CHC sample), and the Research Ethics Boards of the Hospital for Sick Children and St. Michael’s Hospital (non-CHC, *TARGet Kids!*).

### Measures

Parents completed the study instruments, including one based on the Canadian Community Health Survey, [23] addressing child and family characteristics, child diet and health behaviours and the NutriSTEP® questionnaire, a 17-item validated nutritional risk assessment tool [24, 25]. Child weight and height were assessed - according to standardized anthropometric protocols [26]. Venous blood was drawn by trained research personnel, at least 30 min after the application of Ametop® to reduce phlebotomy-associated pain. Samples from the CHC group were tested at Kingston General Hospital (www.kgh.on.ca/) for the complete blood count (CBC) and at Mount Sinai Services (www.mountsinaiservices.com/) for serum ferritin and C-reactive protein (CRP) analysis. For the non-CHC group, samples were tested at Mount Sinai Services.

#### Outcome variables

Our main outcome was iron status, measured by serum ferritin (μg/L) in the absence of inflammation, as recommended by the US National Health and Nutrition Examination Survey [27]. A secondary outcome was hemoglobin. Ferritin was used as a continuous and categorical variable (iron deficiency defined as ferritin < 14 μg/L which was the cut-off used in the US National Health and Nutrition Examination Survey).

#### Covariates

We adjusted our analysis for several pre-specified variables previously reported to influence iron status, including age, sex, body mass index z-score (zBMI), [28] total breastfeeding duration [13] (number of months determined by parental response to the question “For how long has your child been breastfed? – classified as 0 months for children who were never breastfed and their current age for those currently breastfeeding), cow’s milk intake [12] (classified as 2 cups or more- yes/no, consistent with nutritional recommendations) and meat and meat-alternatives consumption [14] (determined by the NutriSTEP® question “My child usually eats meat, fish, poultry, or alternatives (alternatives can be eggs, peanut butter, tofu, nuts, and cooked beans, chickpeas and lentils): 1) More than 2 times a day; 2) Two times a day; 3) Once a day; 4) A few times a week; 5) Not at all.”). We also included iron supplementation (including a multivitamin containing iron) as a covariate. Income and education were also included as potential confounding variables, anticipating a difference in these socioeconomic variables between the CHC and non-CHC groups, and with low socioeconomic status previously reported as a risk factor for iron deficiency in early childhood [29].

### Statistical analysis

We performed descriptive statistics for the main outcomes and covariates. Two multivariable models were constructed. We used a linear regression model to assess the association between CHC attendance and serum ferritin level (as a continuous measure). Serum ferritin was log transformed as residual analysis using the untransformed outcome indicated a violation of the normality assumption. The outcome was then back-transformed to aid in interpretation of results. We used a logistic regression model to evaluate the association between CHC attendance and iron deficiency (using serum ferritin level as a binary measure: serum ferritin value < 14 μg/L or ≥ 14 μg/L). Both models were adjusted for all the covariates previously described regardless of statistical significance [30]. All covariates had less than 10% missing data, with the exception of maternal education, which had 24% missing data, and family income, which had 33% missing data. We included maternal education and family income as covariates to reduce bias in the estimate after confirming that both were confounders using the 10% change-in-estimate criterion [31]. Missing data fit the missing-at-random criterion; we used multiple imputation with 50 imputed datasets methods using the fully conditional specification method to analyze these data [32]. Statistical significance was defined as *p* < 0.05. All statistical tests were two-sided. All analyses were conducted using SAS 9.4 (SAS Institute, Cary, NC).

## Results

Of 310 eligible children at KCHC, 143 declined or could not be reached prior to their third birthday, 111 could not be reached (phone numbers not in service, had no phone or did not respond to a phone or text message) and 15 did not attend a scheduled visit. Forty-one parents consented to their children’s participation and phlebotomy was successful in 31, and none had C-reactive protein values > 10 mg/L. A total of 2619 eligible children from the non-CHC sites consented to participate; of these, 1309 had blood assessed for serum ferritin. Of the 1309 children with serum ferritin values, 902 had data on meat consumption (this sample represented an early datacut for the paper by Cox et al. [14]. Of these, 27 children had C-reactive Protein levels ≥10 mg/L and were excluded. Therefore, there were 875 children from the non-CHC sites who met the inclusion criteria. Previous TARGet Kids! work has found those who opt to have blood sampled are similar in demographics to those who do not [13].

The CHC (*n* = 31) and non-CHC (*n* = 875) samples with blood results differed in their sociodemographic characteristics and health behaviours (Table 1). The CHC sample was older, had lower household income, lower maternal education and differed in maternal ethnicity. The CHC sample had higher nutritional risk as assessed by the NutriSTEP®, greater frequency of intake of cow’s milk exceeding two cups daily and shorter median duration of breastfeeding. Furthermore, growth parameters differed between the two groups; the CHC group had a higher prevalence of both underweight and overweight or obese children compared with the non-CHC cohort. Hematological results in the CHC cohort compared with the non-CHC cohort were hemoglobin (median, range) 121 g/L (99–140) vs. 121 g/L (78–147), ferritin 28 μg/L (4–202) vs. 25 μg/L (2–159), iron deficiency (ferritin< 14 μg/L) 9.7% vs. 18.1%, iron deficiency anemia (Hb < 110 g/L, ferritin< 14 μg/L) 3.2% vs. 2.4%.

**Table 1:** Baseline characteristics of study cohort by exposure variable: CHC attendance

Variable	Total	Attends CHC^a^	Does not attend CHC^a^
Total sample	906	31 (3.4)	875 (96.6)
Age (months)	19.5 (12.2, 36.5)	26.2 (12.9, 36.4)	19.2 (12.2, 36.5)
Sex, male	481 (53.1)	14 (45.2)	467 (53.4)
Maternal Ethnicity			
European	623 (68.8)	22 (71.0)	601 (68.7)
Asian	165 (18.2)	1 (3.2)	164 (18.7)
African/Caribbean/ Latin American	68 (7.5)	3 (9.7)	65 (7.4)
Indigenous	4 (0.4)	2 (6.5)	2 (0.2)
Missing	46 (5.1)	3 (9.7)	43 (4.9)
Child immigration status			
Canadian	858 (94.7)	31 (100)	827 (94.5)
Landed immigrant	20 (2.2)	0	20 (2.3)
Missing	28 (3.1)	0	28 (3.2)
Self-report family income			
$0–29,999	54 (6.0)	19 (61.3)	35 (4.0)
$30,000-79,999	85 (9.4)	6 (19.4)	79 (9.0)
≥ $80,000	469 (51.8)	2 (6.5)	467 (53.4)
Missing	298 (32.9)	4 (12.9)	294 (33.6)
Maternal education			
High school or less	83 (9.2)	20 (64.5)	63 (7.2)
Post-secondary	603 (66.6)	10 (32.3)	593 (67.8)
Missing	220 (24.3)	1 (3.2)	219 (25.0)
Daily iron supplementation, yes	35 (3.9)	3 (9.7)	32 (3.7)
Missing	3 (0.3)	0	3 (0.3)
Daily cow’s milk intake (cups)	2.0 (0, 5)	3.0 (0, 5)	2.0 (0, 5)
Daily cow’s milk intake > 2 cups	278 (30.7)	17 (54.8)	261 (29.8)
Missing	93 (10.3)	2 (6.5)	91 (10.4)
NutriSTEP® total score	12 (1, 39)	17 (6, 34)	12 (1, 39)
Missing	208 (23.0)	1 (3.2)	207 (23.7)
BMI			
Underweight (*z* < −2)	23 (2.5)	2 (6.5)	21 (2.4)
Healthy weight (− 2 ≥ *z* ≤ 1)	682 (75.3)	21 (67.7)	661 (75.5)
At risk overweight (1 > *z* ≤ 2)	124 (13.7)	5 (16.1)	119 (13.6)
Overweight (2 < z ≤ 3)	42 (4.6)	3 (9.7)	39 (4.5)
Obese (*z* > 3)	4 (0.4)	0	4 (0.5)
Missing	31 (3.4)	0	31 (3.5)
Breastfeeding duration, mo	11.0 (0, 35.9) 17 (1.9)	2.0 (0, 23.8) 0	11.0 (0, 35.9) 17 (1.9)
Meat/meat alternatives consumption			
More than twice daily	209 (23.1)	5 (16.1)	204 (23.3)
Twice daily	442 (48.8)	14 (45.2)	428 (48.9)
Once daily	186 (20.5)	7 (22.6)	179 (20.5)
A few times weekly	62 (6.8)	2 (6.5)	60 (6.9)
Not at all	6 (0.7)	2 (6.5)	4 (0.5)
Missing	1 (0.1)	1 (3.2)	0
CRP (mg/L)	0.3 (0.1, 9.7)	0.5 (0.1, 4.8)	0.3 (0.1, 9.7)
Serum ferritin (μg/L)	25 (2, 202)	28 (4, 202)	25 (2, 159)
Iron deficiency, yes (serum ferritin < 14 μg/L)	161 (17.8)	3 (9.7)	158 (18.1)
Hemoglobin (g/L)	121.0 (78, 147)	120.5 (99, 140)	121.0 (78, 147)
Missing	43 (4.8%)	3 (9.7%)	40 (4.6%)
Iron deficiency anemia, yes			
(Serum ferritin < 14 μg/L Hb ≤110 g/L)	21 (2.3)	1 (3.2)	20 (2.3)
Missing	43 (4.8)	3 (9.7)	40 (4.6)

zBMI indicates body mass index z score

^a^Data are presented as n (%) or median (range)

We found no association in the univariate analysis or in the adjusted model between CHC status (yes/no) and iron deficiency (ferritin < 14 μg/L) using multivariable logistic regression (OR 0.55, 95% confidence interval 0.11–2.73, *p* = 0.46) (Table 2). Statistically significant covariates associated with an increased odds of iron deficiency included younger age, higher zBMI, longer total breastfeeding duration, lower meat and meat-alternatives consumption, and lower CRP.

**Table 2:** Multivariable logistic regression model for association between CHC status and iron deficiency (serum ferritin < 14 μg/L)

Predictor	β (95% CI)	OR (CI)	*P*
CHC, yes	− 0.60 (−2.21, 1.01)	0.55(0.11,2.73)	0.46
Age, mo	−0.03 (− 0.06, − 0.001)	0.97(0.94, 1.00)	0.04*
Female sex	− 0.20 (− 0.55, 0.16)	0.82(0.57, 1.17)	0.28
Income			
$0–29,999	0.63 (−0.14, 1.39)	1.88 (0.87, 4.03)	0.84
$30,000-79,999	−0.06 (− 0.69, 0.56)	0.94 (0.50, 1.75)	0.17
≥$80,000 (Reference)			
Maternal education (Post-secondary, no)	−0.57 (−1.37, 0.24)	0.57 (0.25, 1.27)	0.17
zBMI, unit	0.25 (0.09, 0.42)	1.29(1.09, 1.52)	0.004*
Breastfeeding duration, mo	0.04 (0.01, 0.07)	1.04(1.01, 1.08)	0.005*
Milk consumption (> 2 cups daily), yes	0.34 (−0.04, 0.73)	1.41(0.96, 2.08)	0.08
Meat/ meat alternatives consumption	0.21 (−0.41, − 0.005)	0.81(0.66, 0.99)	0.04
CRP (mg/L)	−0.23 (− 0.42, 0.04)	0.79 (0.66, 0.96)	0.02*
Iron/ multivitamin Supplementation, yes	0.01 (−0.92, 0.95)	1.01(0.40, 2.59)	0.98

OR indicates odds ratio; zBMI indicates body mass index z score; CI indicates confidence interval; CRP indicates C-reactive protein. Adjusted β estimates are reported to 2 decimal points for statistical precision. Negative values indicate a decrease in iron deficiency, positive values indicate an increase in iron deficiency. *indicates statistically significant effects

In the univariate linear regression, CHC status was associated with higher serum ferritin (change in serum ferritin 6.73 μg/L [0.32, 14.76, *p* = 0.04]), however, no association was found in the adjusted multivariable linear regression model (change in serum ferritin of 4.8 μg/L (− 2.5, 14.3, *p* = 0.22) for children enrolled from the CHC) (Table 3). Statistically significant covariates associated with lower serum ferritin included higher zBMI, longer total breastfeeding duration, cow’s milk intake greater than two cups daily, lower CRP, and low family income.

**Table 3:** Multivariable linear regression model for association between CHC status and serum ferritin

Predictor	β (log)	95% CI	Change in serum ferritin, %	Change in median serum ferritin		*P*
				μg/l	95% CI	
CHC, yes	0.17	−0.10, −0.45	19.12	4.78	−2.46, 14.34	0.22
Age, mo	−0.0006	−0.01, 0.01	0.06	0.14	−0.15, 0.18	0.87
Female sex	0.08	0.003, 0.16	8.01	2.00	0.07, 4.25	0.06
Income						
$0–29,999	−0.23	−0.41, − 0.04	−20.17	−5.04	−8.48, − 0.89	0.02*
$30,000-79,999	0.02	−0.11, 0.15	1.97	0.49	−2.69, 4.12	0.77
≥$80,000 (Reference)						
Maternal education (Post-secondary, no)	0.08	−0.09, 0.24	7.93	1.98	02.10, 6.79	0.36
zBMI, unit	−0.07	− 0.10,-0.03	−6.49	−1.62	−2.49, 0.71	0.0006*
Breastfeeding duration, mo	−0.01	−0.02, − 0.01	− 1.46	−0.37	− 0.54, − 0.20	<.0001*
Milk consumption (> 2 cups daily)	− 0.11	−0.20, − 0.02	− 10.37	−2.59	−4.49, − 0.52	0.02*
Meat / fish consumption daily	0.01	− 0.04, 0.06	0.94	0.24	− 0.91, 1.44	0.69
CRP (mg/L)	0.07	0.05, 0.10	7.69	1.92	1.17, 2.70	< 0.0001*
Iron/ multivitamin supplementation	0.04	−0.16, 0.25	4.50	1.13	−3.80, 7.20	0.68

zBMI indicates body mass index z score; CI indicates confidence interval; CRP indicates C-reactive protein

Adjusted β estimates are reported to 2 decimal points for statistical precision. Negative values indicate a decrease in median serum ferritin, positive values indicate an increase in median serum ferritin. *indicates statistically significant effects

## Discussion

In this study of healthy 12–36 month old Canadian children, 9.7% of those attending CHCs had non-anemic iron deficiency versus 18.1% of children receiving care in non-CHC sites. We found that despite lower socioeconomic status, there was no association between CHC attendance and iron status. Instead, feeding practices were significant predictors of iron status. The lower prevalence of iron deficiency among children attending CHCs may be explained by the relative distribution of risk and protective factors. For example, the CHC sample had a higher proportion of children with daily cow’s milk intake > 2 cups/day, lower meat and meat-alternatives consumption, and unhealthy weights, but shorter breastfeeding duration. In addition, the small sample size of the CHC cohort may have been an explanatory factor. The association between social determinants and iron deficiency has been examined by other investigators. Small Canadian regional studies have identified a high prevalence of NAID and IDA in children residing in remote Indigenous communities [33–37]. In the U.S., Europe and New Zealand, several studies have identified an association between social factors (low income, [38–41] race/ ethnicity [38, 39, 42, 43] and immigration status [40]) and iron deficiency. The Rourke Baby Record recommends screening for anemia in high risk infants (low SES, some ethnic groups, low birthweight and premature infants and those with excessive milk intake) [20]. The American Academy of Pediatrics recommends universal screening using hemoglobin and risk assessment, including children of low socioeconomic status and of Mexican American decent. Our findings suggest that risk stratification on the basis of feeding practices, rather than social determinants alone, may be a more appropriate strategy for early identification of iron deficiency, a hypothesis which should be empirically tested. Given the high overall prevalence of iron deficiency in this age group, this strategy may be warranted in both CHC and non-CHC settings.

Studies of children with iron deficiency have been largely conducted in developing countries due to the high prevalence. Poor developmental outcomes have been summarized in several reviews of observational studies [1, 44, 45]. A longitudinal study of Costa Rican infants enrolled at 12–23 months and followed to 25 years, showed that the developmental disadvantage persists long term despite iron therapy [46, 47]. These include cognitive impairments (cognitive scores 9 points lower compared with healthy controls) and functional impairments such as non-completion of secondary school and poor emotional health [46, 47]. In addition, case-series and case-control studies suggest an association between iron deficiency anemia and childhood stroke [48, 49].

Extensive evidence has established that biological, physical and social exposures during early childhood have been associated with long term adult health effects, [50–55] but little data exist on the health effects of child poverty in the Canadian context. CHCs may represent an untapped potential to better understand the health needs of vulnerable children.

Strengths of our study include that it is the first published report of the nutritional health of children at Canadian CHCs, as well as the large size of the non-CHC cohort. The CHC model is intended to target socially vulnerable patients [56]. This research provides a preliminary view of the social and nutritional vulnerability of this population and supports the importance of additional research to better understand their health needs, [19] in order to inform the development of interventions aimed at reducing health disparities.

The small size of the CHC cohort is an important study limitation, which limits our power to detect a meaningful difference that may exist. Recruitment in the CHC setting was challenging, despite steps taken to try to reduce barriers to participation including providing transportation, childcare, snacks and a small incentive. Many potential participants could not be reached because their contact information was out of date or they had no telephone, demonstrating the financially precarious situation for many families. Future recruitment may be more successful if undertaken during regularly scheduled clinic visits. Alternatively, CHC staff could be engaged in recruitment and data collection, using research funds for cost recovery, with careful attention to minimizing disruption of clinic workflow. A larger study is needed to address the research questions, to optimize potentially modifiable risk factors for poor developmental outcomes. In addition, our results may be subject to recall bias. It is not known whether recall errors would differ between the two groups.

## Conclusion

Children receiving care at Ontario CHCs have higher social and nutritional risk but we did not identify differences in iron status compared with non-CHC children. Further research is needed to understand the health implications of social adversity in general, and iron deficiency in particular, in the CHC cohort.

## Abbreviations

CBC: Complete blood count
CHC: Community Health Centre
CRP: C-reactive protein
IDA: Iron deficiency anemia
NAID: Non-anemic iron deficiency
z BMI: Body mass index z-score
